# Supplementary material for: Germ granule compartments coordinate specialized small RNA production
Source: Nat Commun. 2024 Jul 10;15:5799. doi: 10.1038/s41467-024-50027-3 (PMC11236994; doi:10.1038/s41467-024-50027-3)
Supplement: Supplementary file 1 — Supplementary Information [file 41467_2024_50027_MOESM1_ESM.pdf]

Supplementary Information for

Germ granule compartments coordinate specialized small RNA production

Xiangyang Chen[1ǂ], Ke Wang[1ǂ], Farees Ud Din Mufti[1ǂ], Demin Xu[1], Chengming Zhu[1], Xinya Huang[1], Chenming Zeng[1], Qile Jin[1], Xiaona Huang[1], Yong-hong Yan[2], Meng-qiu Dong[2], Xuezhu Feng[3*], Yunyu Shi[1*], Scott Kennedy[4*], and Shouhong Guang[1,5*]

[1]Department of Obstetrics and Gynecology, The First Affiliated Hospital of USTC, The USTC RNA Institute, Ministry of Education Key Laboratory for Membraneless Organelles & Cellular Dynamics, Hefei National Research Center for Physical Sciences at the Microscale, Center for Advanced Interdisciplinary Science and Biomedicine of IHM, School of Life Sciences, Division of Life Sciences and Medicine, Biomedical Sciences and Health Laboratory of Anhui Province, University of Science and Technology of China, Hefei, Anhui 230027, China

[2]National Institute of Biological Sciences, Beijing 102206, China.

[3]School of Basic Medicine, Anhui Medical University, Hefei, China

[4]Department of Genetics, Blavatnik Institute at Harvard Medical School, Boston, MA 02115, USA.

[5]CAS Center for Excellence in Molecular Cell Science, Chinese Academy of Sciences, Hefei, Anhui 230027, P.R. China

ǂThese authors contributed equally to this work.

*Correspondence should be addressed to fengxz@ustc.edu.cn, yyshi@ustc.edu.cn, kennedy@genetics.med.harvard.edu, and sguang@ustc.edu.cn

a

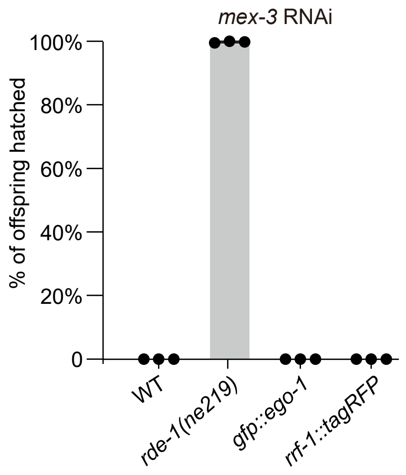

b

Strain	Control RNAi	*unc-15* RNAi
WT	-	+++++
rde-1(ne219)	-	-
rrf-1(pk1417)	-	-
rrf-1::tagRFP	-	+++++

c

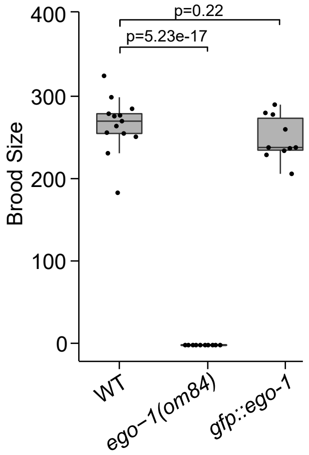

d

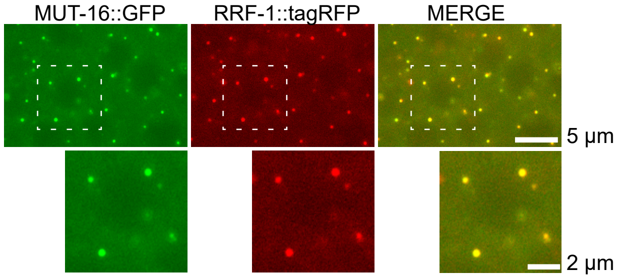

Supplementary Fig. 1. Tagging EGO-1 and RRF-1 with fluorescent labels does not affect their cellular functions. (a-c) Addition of epitope tags by CRISPR/Cas9-mediated gene conversion of *ego-1* or *rrf-1* did not disrupt the functions of tagged proteins in feeding RNAi response and fertility. (a) Quantification of hatched embryos after feeding RNAi targeting *mex-3*. Synchronized L1 stage animals of the indicated genotypes were cultured on plates seeded with bacteria expressing *mex-3* dsRNA. The numbers of total F1 embryos and hatched F1 embryos were scored. Data are presented as the mean +/-SD of three biologically independent samples. (b) Score of the movement ability of animals of the indicated genotypes after feeding RNAi targeting *unc-15*. Synchronized L1 stage animals of the indicated genotypes were cultured on plates seeded with bacteria expressing *unc-15* dsRNA. The movement ability of adult-stage worms was scored. The silencing effects of *unc-15* RNAi are roughly denoted as '+++++' (silencing) and '-' (no silencing), as described in a previous study 1. n = 3 biologically independent samples. (c) Brood size of the indicated animals. L3 animals are singled to individual NGM plates seeded with OP50 bacteria. Brood sizes are scored (N≥10 animals). Bolded midline indicates median value, box indicates the first and third quartiles, and whiskers represent the most extreme data points within 1.5 times the interquartile range. A two-tailed *t*-test was performed to determine statistical significance. The *ego-1(om84)* allele was balanced by a *hT2[bli-4(e937) let-?(q782) qIs48] (I;III)* chromosome. Homozygous *ego-1(om84)* animals were selected by picking animals without pharyngeal GFP under a fluorescence stereomicroscope (Leica) when they reached the L4 stage. (d) RRF-1 localizes to Mutator foci. Pachytene germ cells of animals that express RRF-1::tagRFP and MUT-16::GFP were imaged. Images are representative of more than three animals. Source data are provided as a Source Data file.

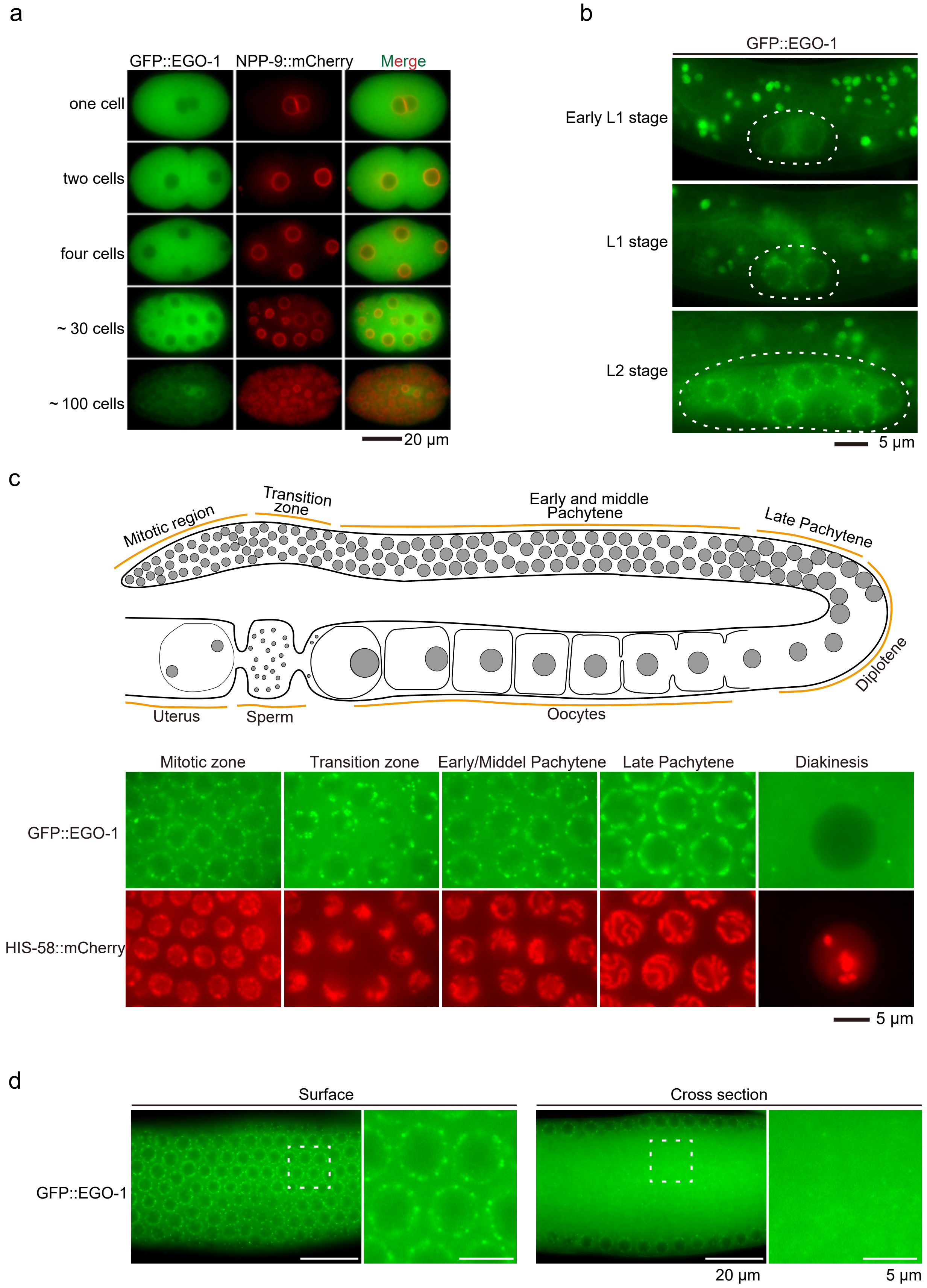

Supplementary Fig. 2. EGO-1 accumulates in perinuclear foci in germ cells and largely diffuses in the cytosol during diakinesis and embryogenesis.(a) Embryos of animals that express GFP::EGO-1 and NPP-9::mCherry. Adult animals were dissected, and embryos were imaged. (b) Live imaging of GFP::EGO-1 in representative larval stages. Bleached embryos were placed onto bacteria-free NGM plates and cultured for 10 hours. Newly hatched L1 stage (early L1 stage) worms were transferred to NGM plates seeded with OP50 bacteria to allow growth. After feeding with OP50 for 6-8 hours, the L1 stage worms were imaged. (c) Fluorescence micrographs of the germ cells of adult *gfp::ego-1;his-58::mCherry* animals at different differentiation stages. Upper, schematic representation of the *C. elegans* adult hermaphrodite germ line. Bottom, fluorescence micrographs of the germ cells at different stages. (d) Fluorescence micrographs of the surface and rachis of the germline in live adult animals expressing GFP::EGO-1. All images are representative of more than three animals.

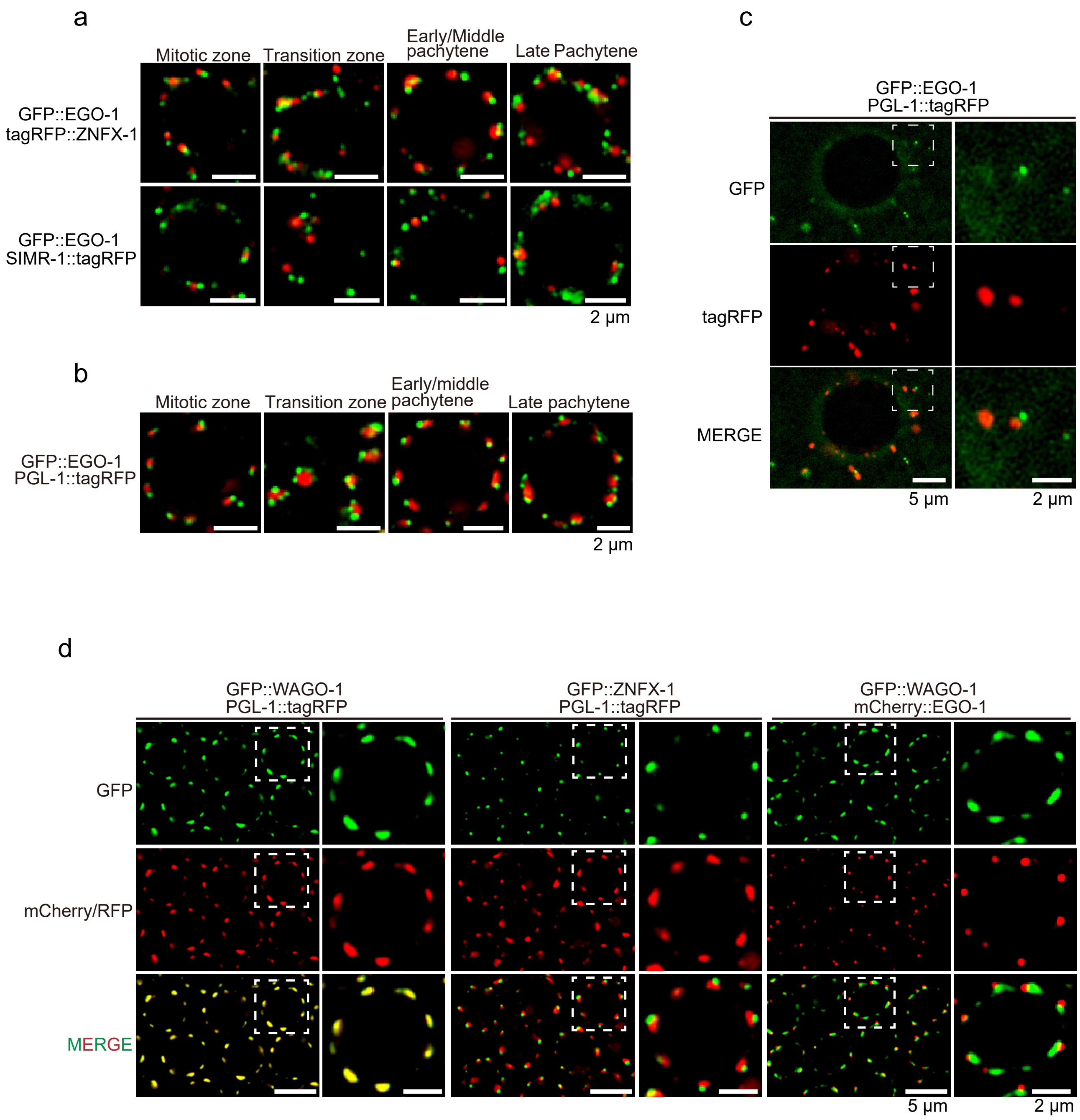

Supplementary Fig. 3. EGO-1 foci in germ cells do not colocalize with ZNFX-1, SIMR-1, PGL-1 or WAGO-1. (a) Fluorescence micrographs of germ cells of animals that express GFP::EGO-1 and indicated tagRFP-tagged proteins. EGO-1 foci do not colocalize with Z or S compartments of the germ granule throughout the germline. (b) Fluorescence micrographs of germ cells of animals that express GFP::EGO-1 and PGL-1::tagRFP. Germlines from dissected adult stage worms were imaged. Multiple EGO-1 foci usually attach to a single P granule in the transition zone and late pachytene stage. (c) Fluorescence micrographs of an oocyte of animals that express GFP::EGO-1 and PGL-1::tagRFP. (d) Fluorescence micrographs of the early/mid pachytene germ cells of animals that express the indicated proteins. All images were acquired with a Leica THUNDER Imaging System and deconvoluted using Leica Application Suite X software (Version 3.7.4.23463). All images are representative of more than three animals.

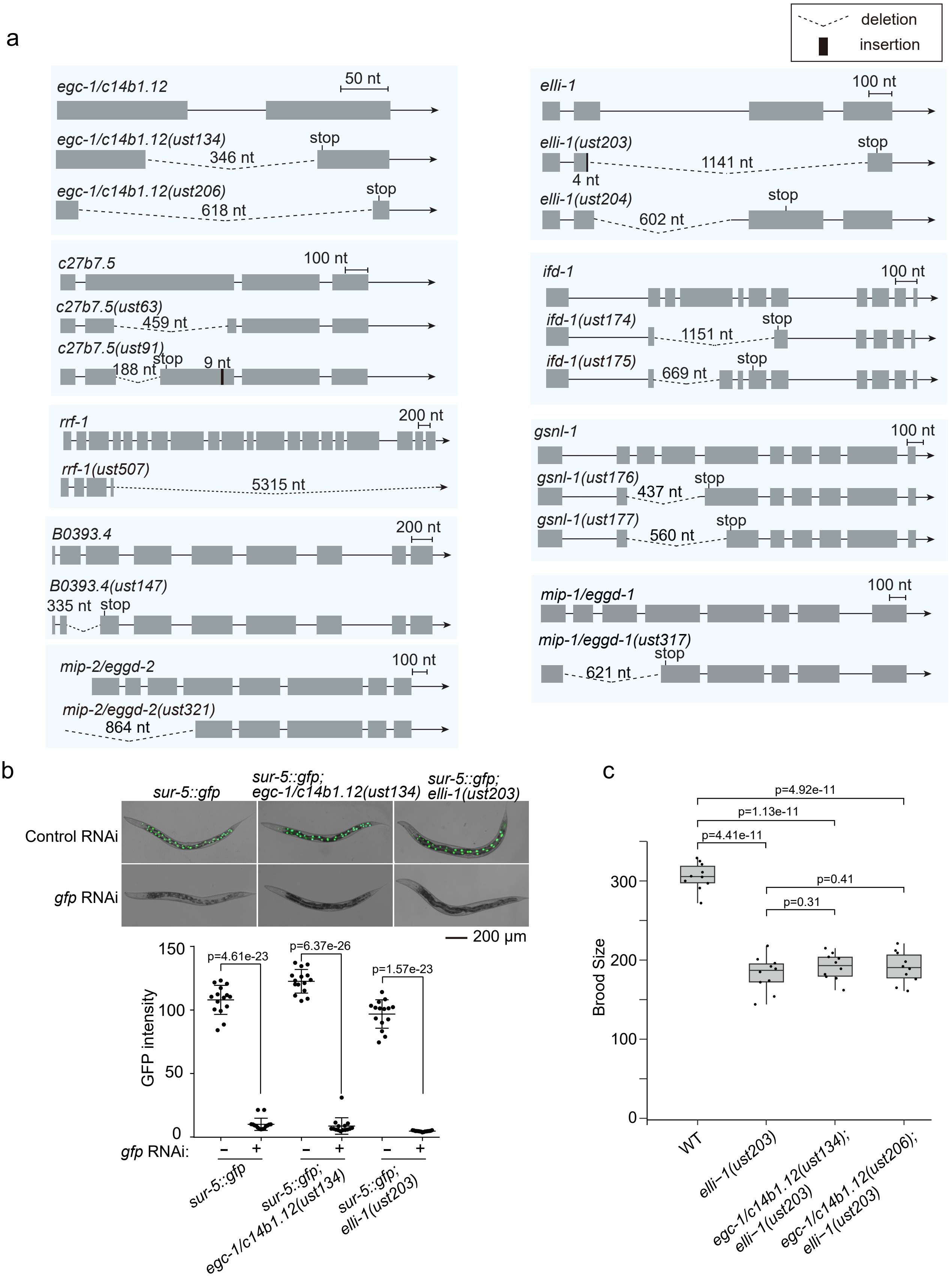

Supplementary Fig. 4. Sequence information of mutants generated in this study using CRISPR/Cas9 technology and the loss of EGC-1 or ELLI-1 does not affect somatic RNAi. (a) Sequence information of alleles generated using a multiple sgRNA-based CRISPR/Cas9 gene editing system [1]. Deletions and insertions are to scale. Premature termination codons caused by frameshift mutations are indicated. (b) The loss of EGC-1 or ELLI-1 does not affect somatic RNAi. Animals expressing a *sur-5::gfp* transgene were exposed to *gfp* RNAi. Bleached *sur-5::gfp* embryos were cultured on RNAi plates seeded with bacteria expressing *gfp* dsRNA. Somatic cells at the young adult stage were imaged. Left: Fluorescence images of the indicated animals without or with *gfp* RNAi. Right: The GFP intensity levels in the indicated animals were measured with ImageJ. Fifteen animals in each group were used for measurement. Data are presented as the mean +/-SD. A two-tailed *t*-test was performed to determine statistical significance. (c) Brood sizes of the indicated animals at 20 ℃. Bleached embryos were hatched and grown at 20 ℃. Then, L3 worms were transferred individually onto fresh NGM plates. The number of progeny worms was scored. N=10 animals. Bolded midline indicates median value, box indicates the first and third quartiles, and whiskers represent the most extreme data points within 1.5 times the interquartile range. A two-tailed *t*-test was performed to determine statistical significance. Source data are provided as a Source Data file.

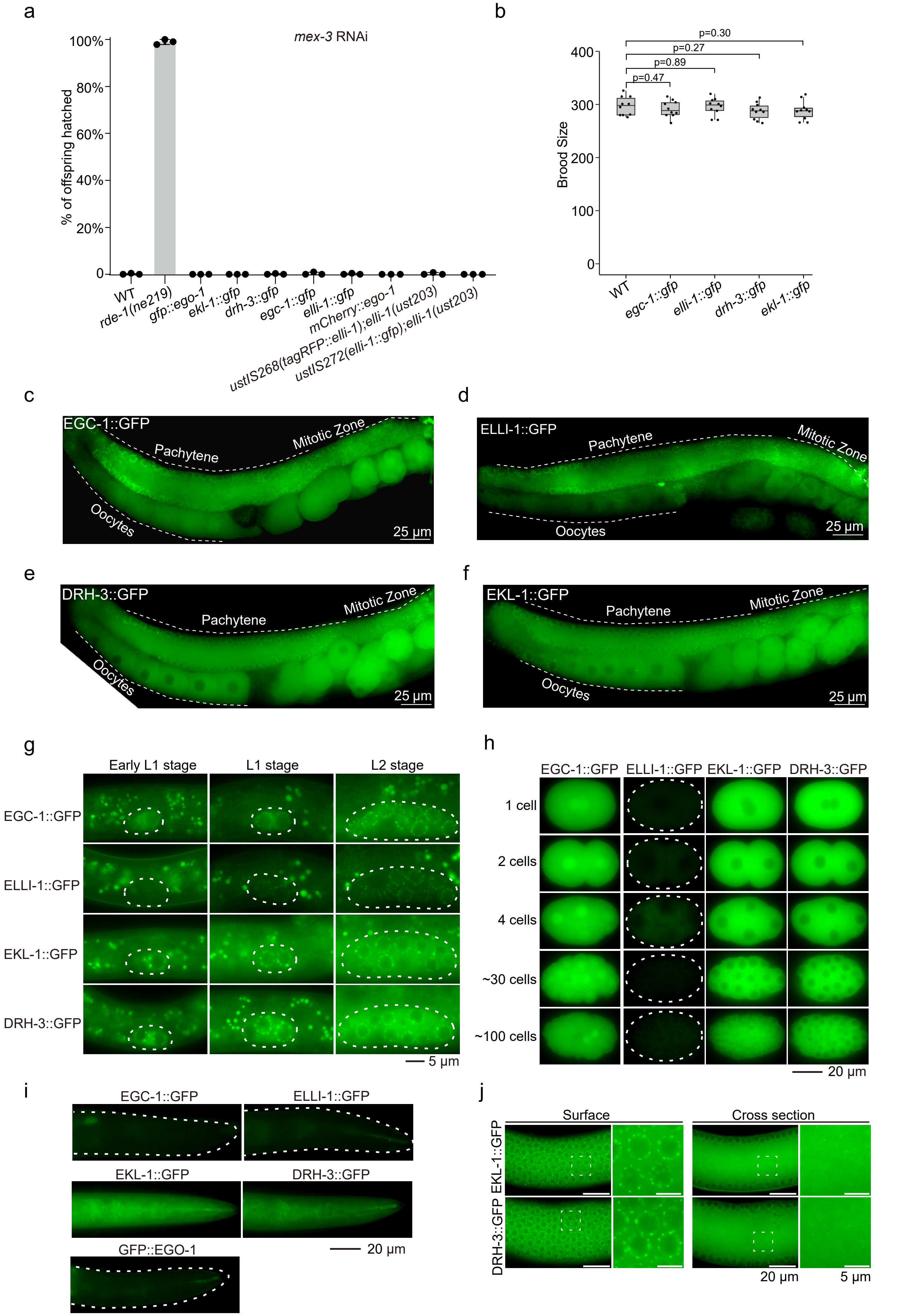

Supplementary Fig. 5. The expression patterns of EGC-1, ELLI-1, EKL-1 and DRH-3. (legend continued on next page)

Supplementary Fig. 5. The expression patterns of EGC-1, ELLI-1, EKL-1 and DRH-3. (a, b) Tagging EGC-1, ELLI-1, DRH-3 and EKL-1 with fluorescent labels does not affect their functions in feeding RNAi (a) and fertility (b). (a) Quantification of hatched embryos after feeding RNAi targeting *mex-3*. Synchronized L1 stage animals of the indicated genotypes were cultured on plates seeded with bacteria expressing *mex-3* dsRNA. The numbers of total F1 embryos and hatched F1 embryos were scored. Data are presented as the mean+/-SD of three biologically independent samples. (b) Brood size of the indicated animals. L3 animals are singled to individual NGM plates seeded with OP50 bacteria. Brood sizes are scored (N≥10 animals). Bolded midline indicates median value, box indicates the first and third quartiles, and whiskers represent the most extreme data points within 1.5 times the interquartile range. A two-tailed *t*-test was performed to determine statistical significance. Source data are provided as a Source Data file. (c-f) Fluorescence micrographs of animals expressing EGC-1::GFP (c), ELLI-1::GFP (d), DRH-3::GFP (e) or EKL-1::GFP (f). Images were acquired using a 40× objective. (g) Live imaging of EGO-1 interactors at representative larval stages. Bleached embryos were placed onto bacteria-free NGM plates and cultured for 10 hours. Newly hatched L1 stage (early L1 stage) worms were transferred to NGM plates seeded with OP50 bacteria to allow growth. In early L1 stage animals, EGC-1::GFP diffused in germ cells; ELLI-1::GFP were barely expressed; EKL-1 and DRH-3::GFP accumulated to perinuclear foci, which may be Mutator foci. After feeding with bacteria for 6-8 hours, the five proteins are continuously expressed and accumulate in perinuclear foci throughout larval development. (h) Fluorescence micrographs of animals expressing EGC-1::GFP, ELLI-1::GFP, EKL-1::GFP or DRH-3::GFP in early embryos. During embryogenesis, EGC-1::GFP is enriched in the nucleus and also diffused in the cytosol; ELLI-1::GFP was hardly detectable; EKL-1::GFP and DRH-3::GFP diffused in the cytosol but were excluded from the nucleus. (i) Live imaging of the heads of animals expressing the indicated fluorescent proteins at the larval stage. (j) Fluorescence micrographs of the surface and rachis of the germline in live adult animals expressing EKL-1::GFP or DRH-3::GFP. All images are representative of more than three animals.

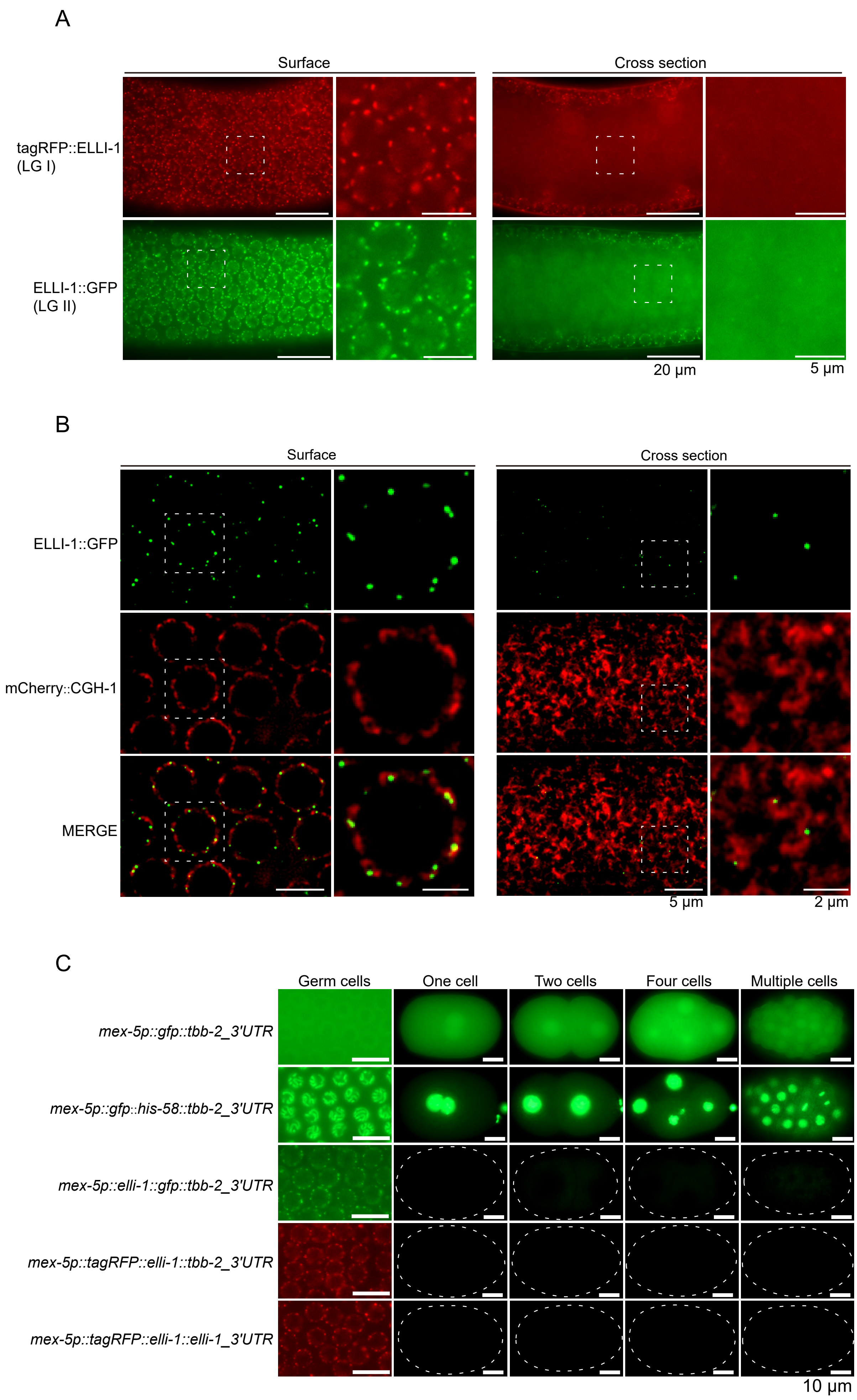

Supplementary Fig. 6. ELLI-1 is expressed in the germline, and mainly localizes to the surface of the germline.
(legend continued on next page)

Supplementary Fig. 6. ELLI-1 is expressed in the germline, and mainly localizes to the surface of the germline.

(a) Fluorescence micrographs of the surface and rachis of the germline in live adult animals expressing ectopic tagRFP::ELLI-1(*ustIS268*, IG I) or ELLI-1::GFP(*ustIS272*, LG II). Both tagRFP::ELLI-1(*ustIS268*, IG I) and ELLI-1::GFP(*ustIS272*, LG II) largely accumulated in the perinuclear region. Images were taken by a Leica upright DM4B microscope equipped with a Leica DFC7000 T camera. (b) Fluorescence micrographs of animals expressing ELLI-1::GFP(*ust374*, *in situ*) and mCherry::CGH-1. CGH-1 is a marker of the P-body, which forms a coating around the P granule [2]. ELLI-1::GFP(*ust374*, *in situ*) localizes to germline foci that do not colocalize with mCherry::CGH-1. Images were acquired with the Leica THUNDER Imaging System and deconvoluted using Leica Application Suite X software (Version 3.7.4.23463). (c) Fluorescence micrographs of pachytene germ cells and early embryos from animals expressing the indicated fluorescent proteins. The ectopic *mex-5p::gfp::tbb-2_3'UTR* element was inserted into LG II. Subsequently, the transgene carrying the *mex-5p::elli-1::gfp::tbb-2_3UTR* element was constructed by inserting an *elli-1* protein coding sequence immediately upstream of the *gfp* start codon. The ectopic *mex-5p::tagRFP::elli-1::tbb-2_3'UTR* and *mex-5p::tagRFP::elli-1::elli-1_3'UTR* were inserted into the *ttTi4348* locus on LG I. Images were taken by a Leica upright DM4 B microscope equipped with a Leica DFC7000 T camera. Images are representative of at least two independent lines of each transgene.

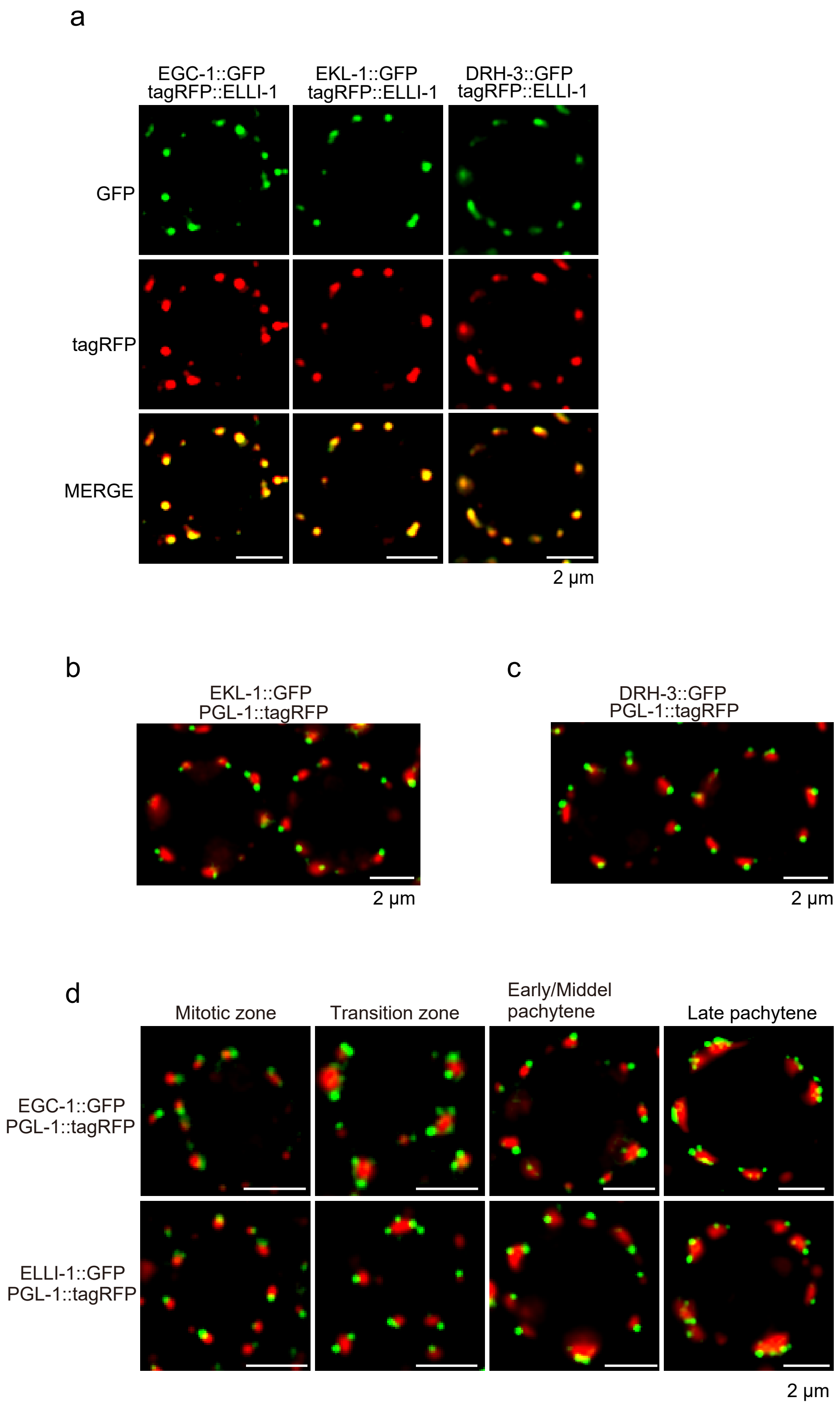

Supplementary Fig. 7. EGO-1 interactors localize to the perinuclear condensates attached to the P granule. (a) Pachytene germ cells of animals that express tagRFP::ELLI-1 and the indicated GFP-tagged proteins. (b, c) Pachytene germ cells of animals that express EKL-1::GFP and PGL-1::tagRFP (b), and DRH-3::GFP and PGL-1::tagRFP (c). (d) Fluorescence micrographs of animals expressing the indicated proteins during germline development. Similar to EGO-1 foci, multiple EGC-1 foci or ELLI-1 foci generally attach to a single P granule in the transition zone and the late pachytene stage. All images were captured via the Leica THUNDER Imaging System and deconvoluted using Leica Application Suite X software (Version 3.7.4.23463). All images are representative of more than three animals.

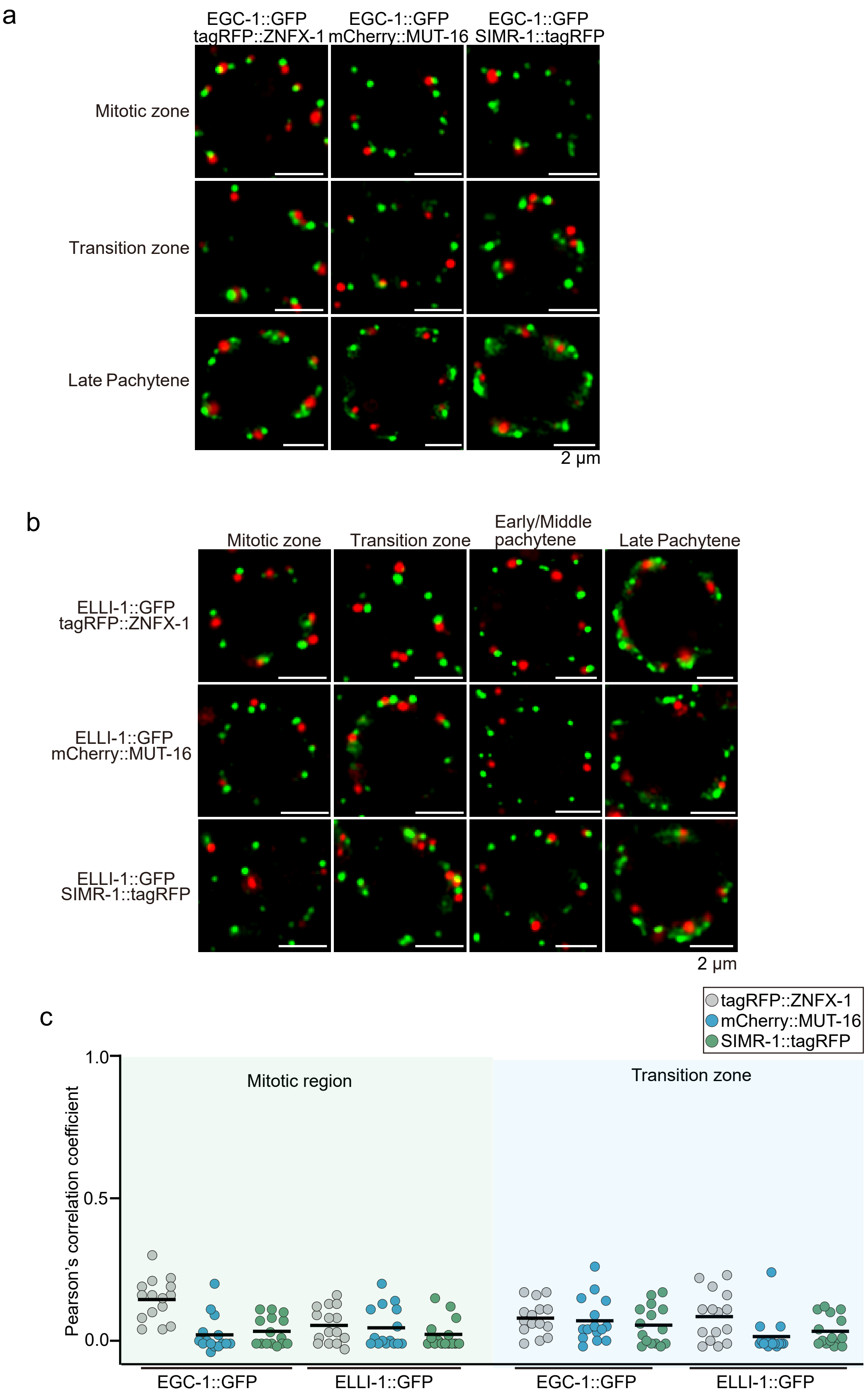

Supplementary Fig. 8. EGC-1 and ELLI-1 do not colocalize with the Z, M or S compartments of the germ granule throughout the germline. (a, b) Fluorescence micrographs of the germ cells of adult animals that express EGC-1::GFP (a), or ELLI-1::GFP (b), and indicated tagRFP-labeled proteins at different differentiation stages of germ cells. (c) Quantification of colocalization between the indicated fluorescent proteins of germ cells in the mitotic region and transition zone. Each data point represents the Pearson's R value showing the degree of colocalization between two fluorescence channels covering an individual germ cell (15 germ cells in total from 3 independent animals). The solid black line indicates the mean value. All images were captured via the Leica THUNDER Imaging System and deconvoluted using Leica Application Suite X software (Version 3.7.4.23463). As the intensities of germ granule compartments intensively vary along the adult germline, the display values of fluorescence images showing the relative position of germ granule compartments with E granule components were manually adjusted to visualize these proteins in different germline regions using Leica Application Suite X software (version 3.7.4.23463). All images are representative of more than three animals.

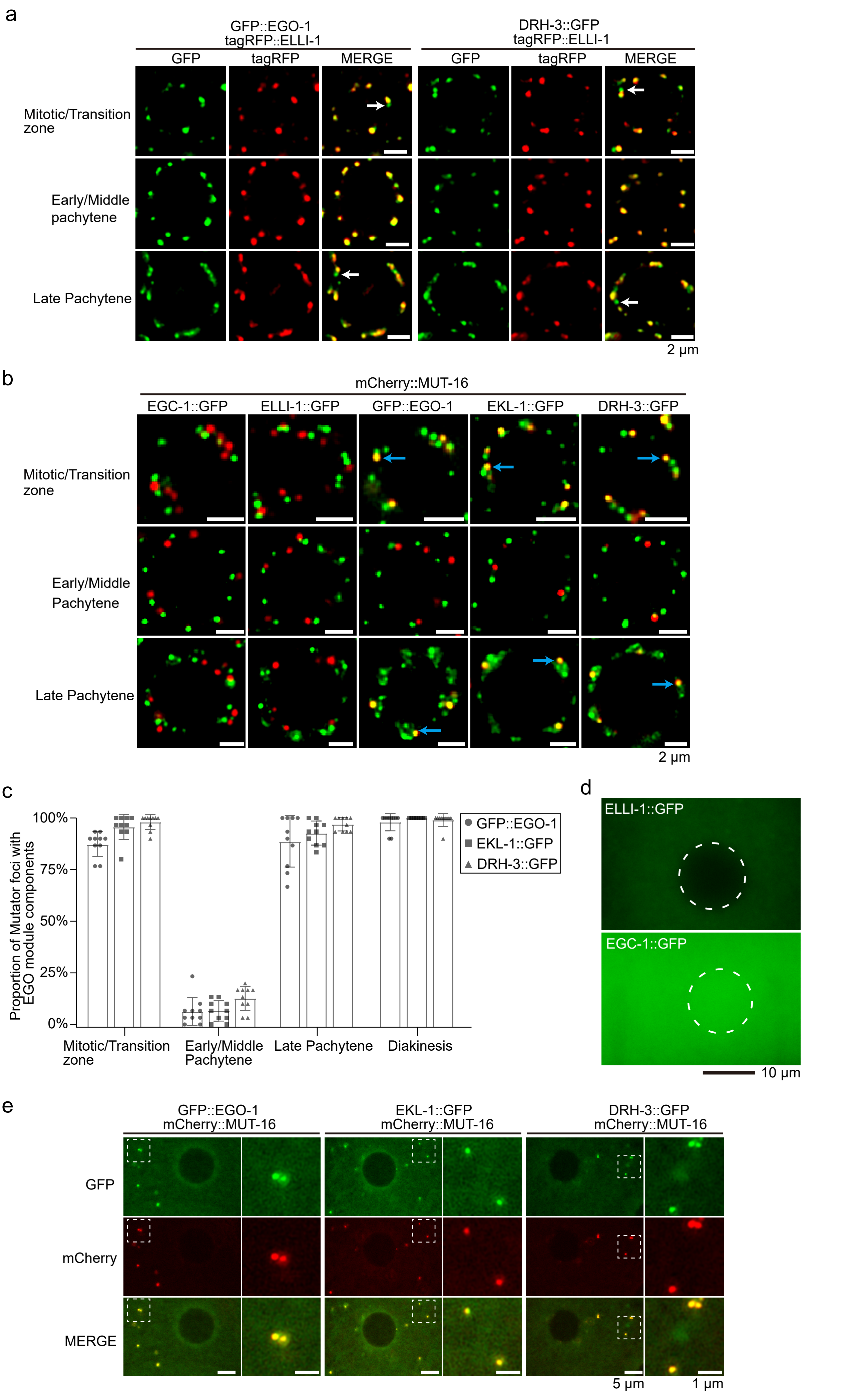

Supplementary Fig. 9. The relative distribution of the EGO module to the E and M compartments of the germ granule varies across the stages of germ cell development. (legend continued on next page)

Supplementary Fig. 9. The relative distribution of the EGO module to the E and M compartments of the germ granule varies across the stages of germ cell development. (a) Fluorescence micrographs of animals expressing tagRFP::ELLI-1 and the indicated GFP-tagged EGO module components (EGO-1 and DRH-3) in different germline regions. The white arrows indicate the EGO foci that do not colocalize with tagRFP::ELLI-1. (b) Fluorescence micrographs of animals expressing mCherry::MUT-16 and the indicated GFP-tagged EGO module components in different germline regions. The EGO module factors partially accumulated in Mutator foci in mitotic cells, the transition zone and the late pachytene of adult germlines. The blue arrows indicate the EGO foci that colocalize with mCherry::MUT-16. The subcellular localization patterns of EGO-1 and its interacting partners are summarized in Fig. 4c. (c) The proportion of Mutator foci containing EGO module components in different regions of the germline. Germlines from 10 adult animals were imaged and analyzed. 10 Mutator foci from the diakinesis region of each animal and 30 Mutator foci from other regions of each animal were selected and analyzed. Data are presented as the mean +/-SD. Source data are provided as a Source Data file.(d) Fluorescence micrographs of animals expressing the indicated GFP-tagged proteins at the end of meiotic prophase, in diakinesis. During diakinesis, ELLI-1 is barely expressed, and EGC-1 diffuses in the nucleus and the cytosol. (e) Fluorescence micrographs of animals expressing mCherry::MUT-16 and the indicated GFP-tagged EGO module components in the diakinesis stage. The EGO module accumulated in Mutator foci during diakinesis. All images were captured via the Leica THUNDER Imaging System and deconvoluted using Leica Application Suite X software (Version 3.7.4.23463). As the intensities of Mutator foci intensively vary along the adult germline, the display values of fluorescence images showing the relative position of Mutator foci with E granule components were manually adjusted to visualize these proteins in different germline regions using Leica Application Suite X software (version 3.7.4.23463). All images are representative of more than three animals.

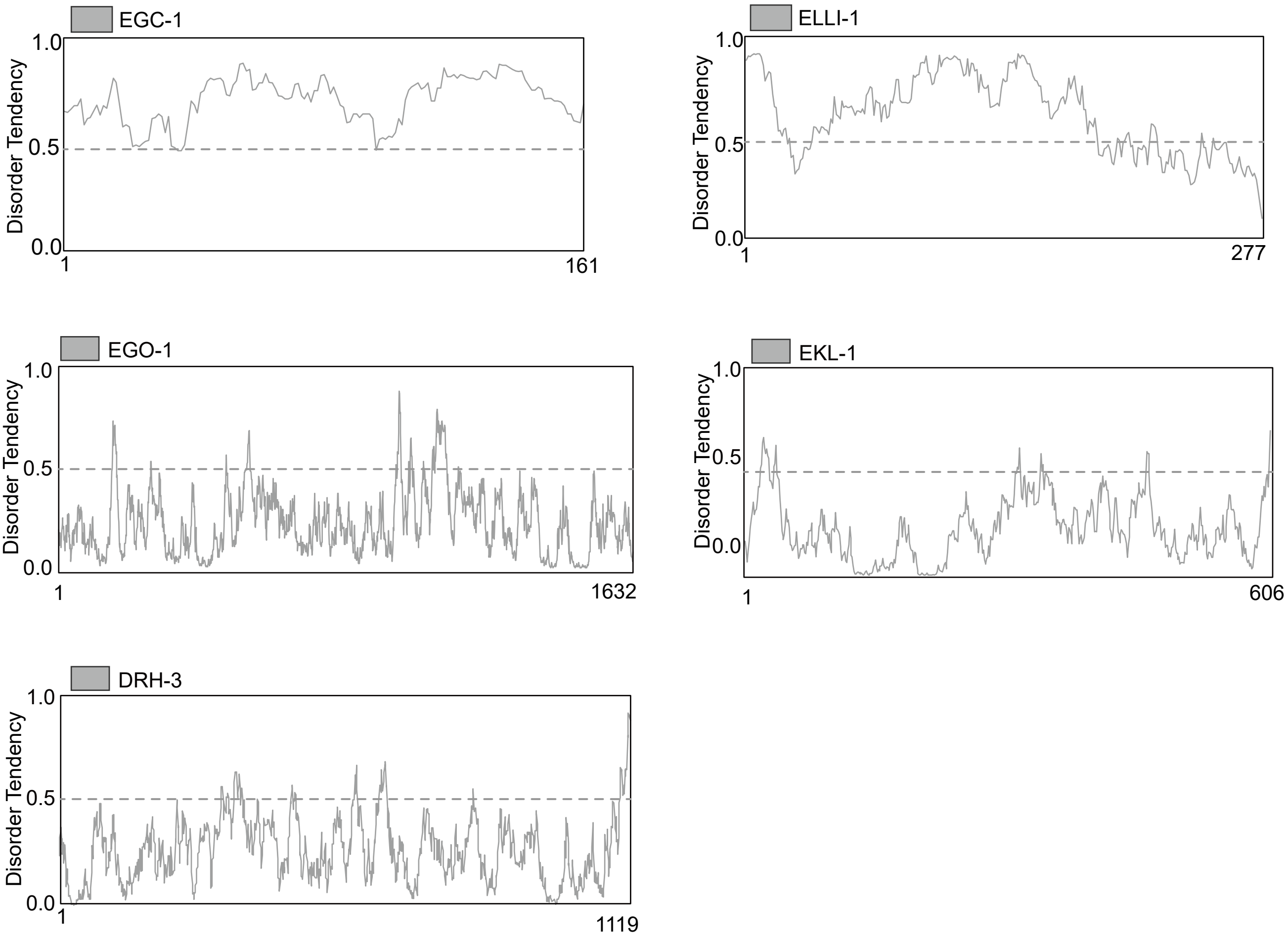

Supplementary Fig. 10. EGC-1 and ELLI-1 orthologs contain a high degree of predicted disorder. Graphs comparing the disordering tendency of *C. elegans* EGO-1 and EGO-1 interactors using the IUPred2A program (https://iupred2a.elte.hu/). IUPred2A provides a score that characterizes the disordered tendency of each position along the sequence [3]. This score can take a value between 0 and 1. Residues with a predicted score above 0.5 are considered disordered,while residues with lower scores are considered ordered.

a

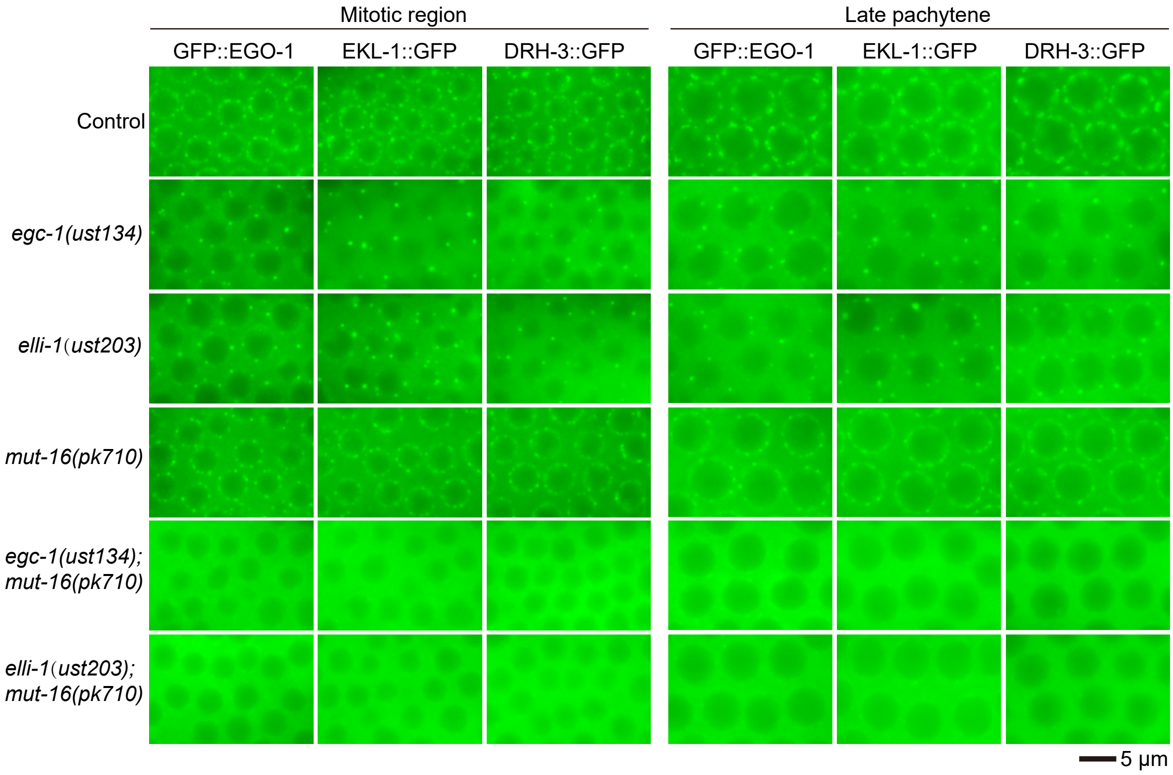

b

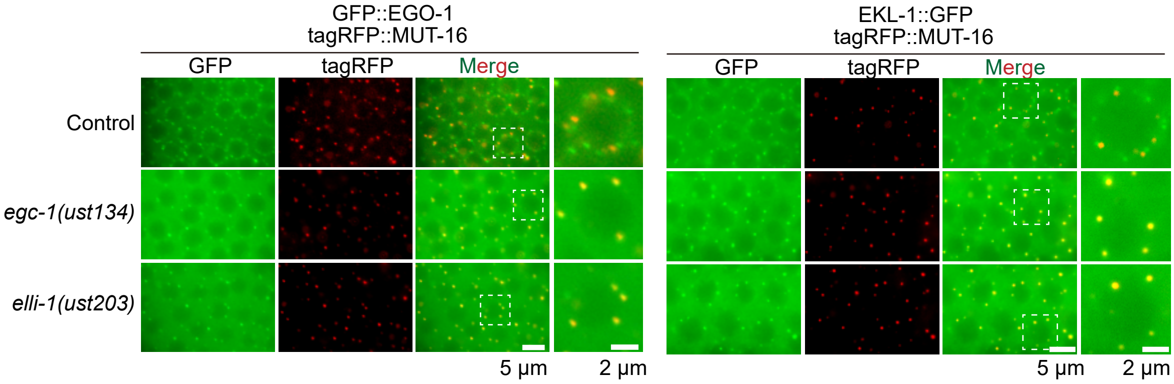

Supplementary Fig. 11. EGC-1, ELLI-1 and MUT-16 promote the perinuclear localization of the EGO module in germ cells. (a) Fluorescence micrographs of the GFP-tagged EGO module components in mitotic cells and germ cells at late pachytene in the indicated animals. (b) Fluorescence micrographs of pachytene cells of animals expressing tagRFP::MUT-16 and GFP::EGO-1, or tagRFP::MUT-16 and EKL-1::GFP in the indicated animals. All images are representative of more than three animals.

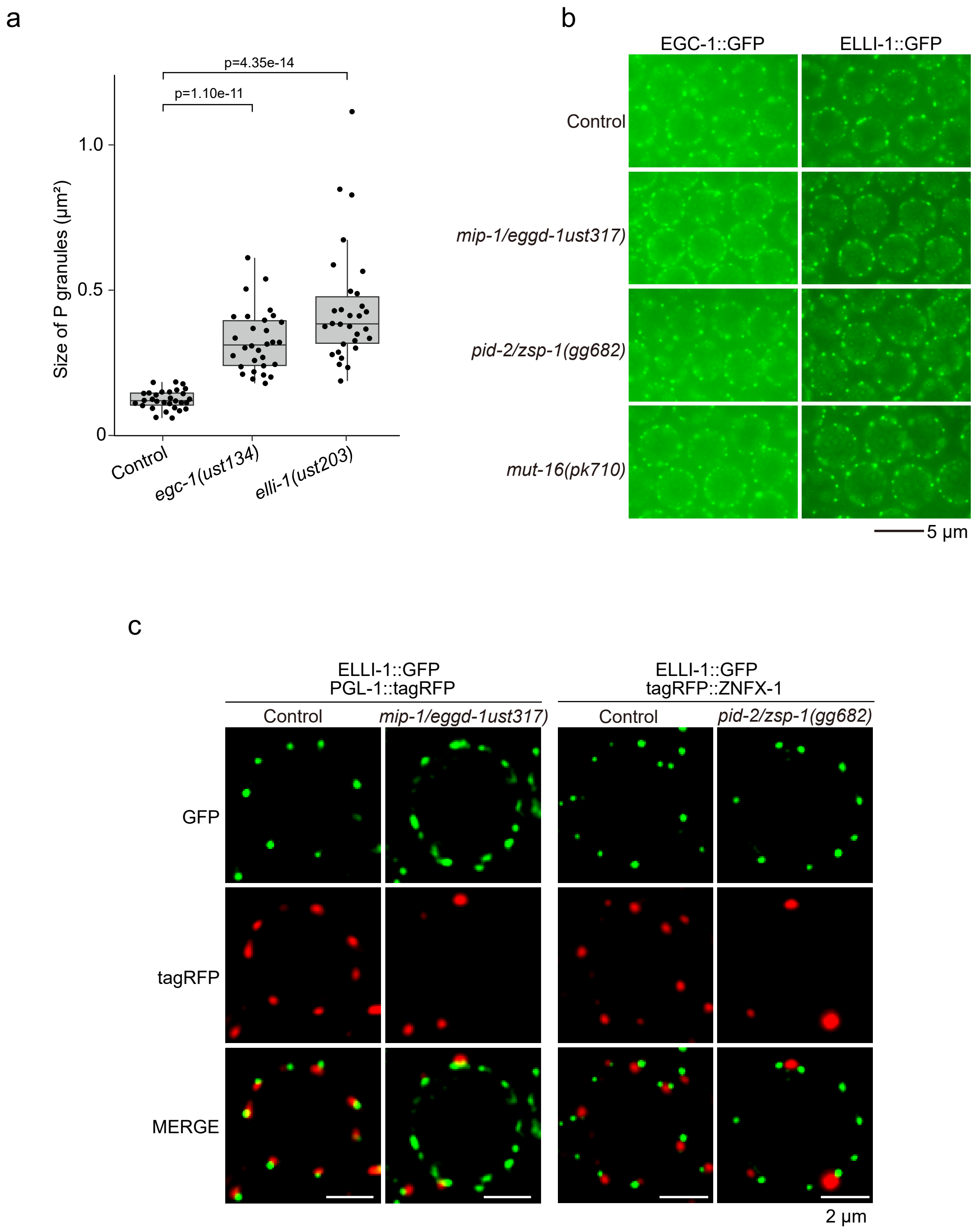

Supplementary Fig. 12. The E granule forms independently of other germ granule compartments. (a) Quantification of the sizes of PGL-1::GFP foci in the indicated mutants. Each data point represents the quantification of an individual focus (30 foci in 3 independent animals). Bolded midline indicates median value, box indicates the first and third quartiles, and whiskers represent the most extreme data points within 1.5 times the interquartile range. A two-tailed *t*-test was performed to determine statistical significance. Source data are provided as a Source Data file. (b) Fluorescence micrograph of EGC-1::GFP and ELLI-1::GFP in the indicated animals. Images were taken by a Leica upright DM4 B microscope equipped with a Leica DFC7000 T camera. (c) Fluorescence micrograph of ELLI-1::GFP and PGL-1::tagRFP, or ELLI-1::GFP and tagRFP::ZNFX-1 in the indicated animals. Images were captured via the Leica THUNDER Imaging System and deconvoluted using Leica Application Suite X software (Version 3.7.4.23463). MIP-1 is required for perinuclear localization of P, Z and M compartments of the germ granule [4, 5, 6]; ZSP-1/PID-2 is a Z granule surface protein that is required for the assembly or morphology of Z granules [7, 8]. The depletion of MIP-1/EGGD-1 or ZSP-1/PID-2 did not affect the size or distribution of perinuclear EGC-1::GFP or ELLI-1::GFP foci. All images are representative of more than three animals.

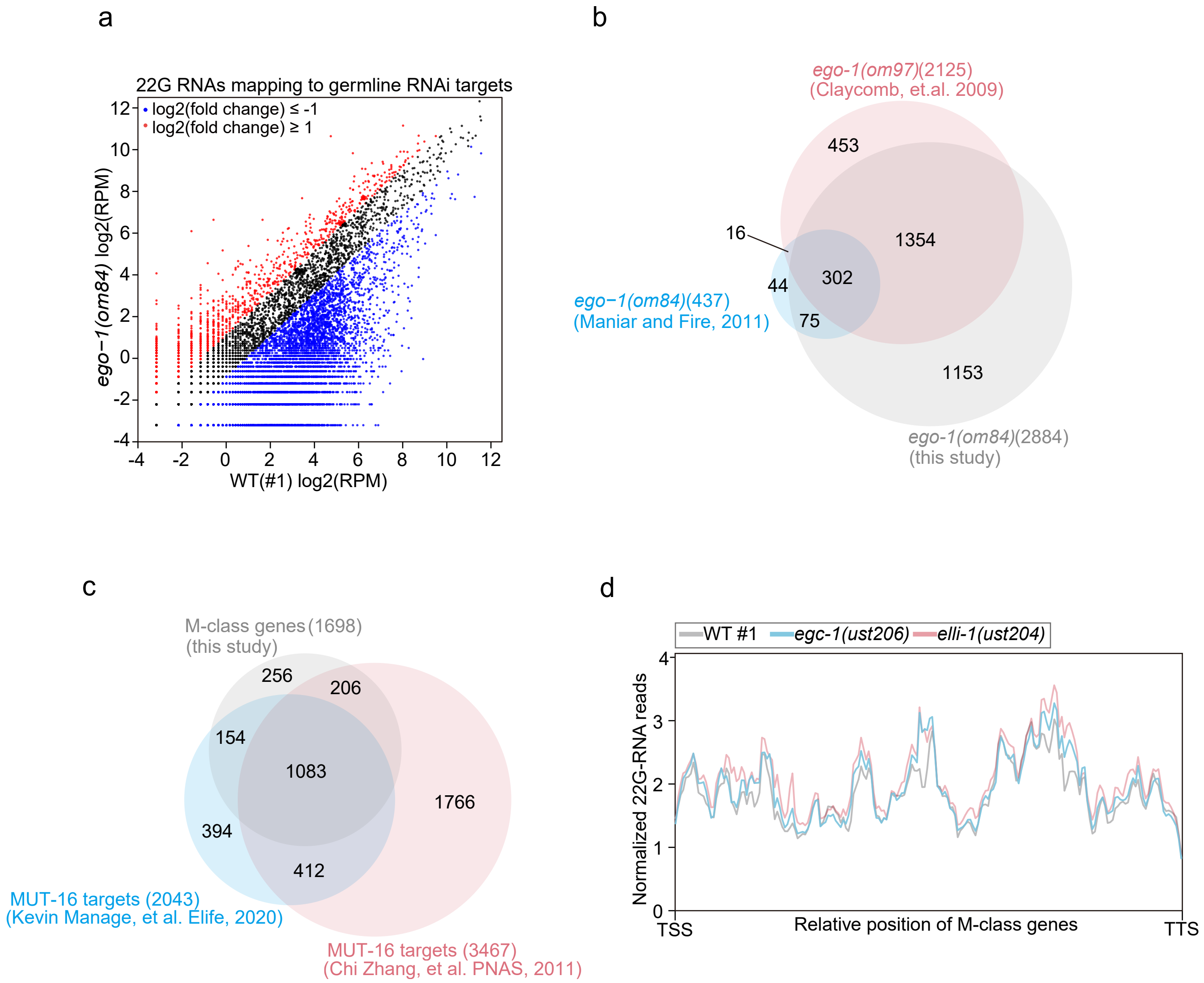

Supplementary Fig. 13. Identification of EGO-1-dependent siRNAs and MUT-16-dependent siRNAs. (a) Scatter plots showing gene-by-gene comparisons of normalized siRNA abundances. siRNAs from wild-type, *ego-1(-)* animals were sequenced. 22G RNAs were mapped to the *C. elegans* genome, and the number of reads complementary to each *C. elegans* gene was quantified. A cutoff criterion of a 2-fold change was applied to identify differentially expressed siRNAs. Genes with upregulated and downregulated siRNAs are shown in red and blue, respectively. (b) Proportional Venn diagram showing comparisons between EGO-1 siRNA targets in the current study and published EGO-1-dependent siRNA targets [9, 10]. Published soma-enriched siRNA targets were excluded [11]. (c) Proportional Venn diagram showing comparisons between M-class genes in the current study and published MUT-16-dependent siRNA targets [12, 13]. Published soma-enriched siRNA targets were excluded [11]. (d) Metaprofile analysis showing the distribution of normalized 22G RNA (sRNA-seq) reads (RPM) along M-class genes in the indicated animals.

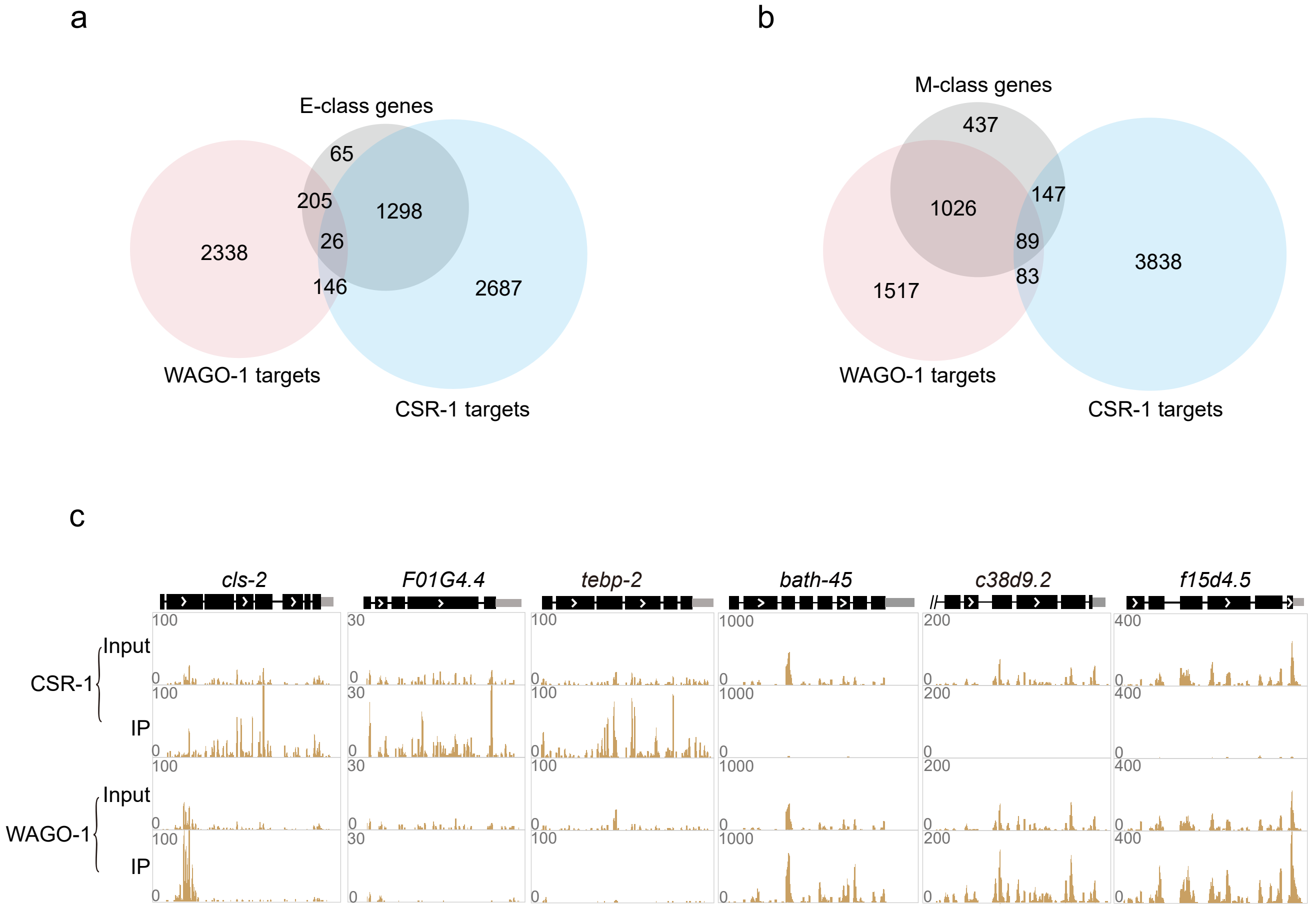

Supplementary Fig. 14. E granules and Mutator foci contribute to the generation of different classes of siRNAs, which bind to distinct Argonautes. (a) Proportional Venn diagram showing the overlap among E-class genes, CSR-1 targets and WAGO-1 targets. The CSR-1 targets and WAGO-1 targets were identified from published papers [10, 11]. (b) Proportional Venn diagram showing the overlap among M-class genes, CSR-1 targets and WAGO-1 targets. (c) Examples of normalized antisense 22G RNA distribution mapping to the indicated genes in the indicated samples. Data from a published paper is analyzed [14].

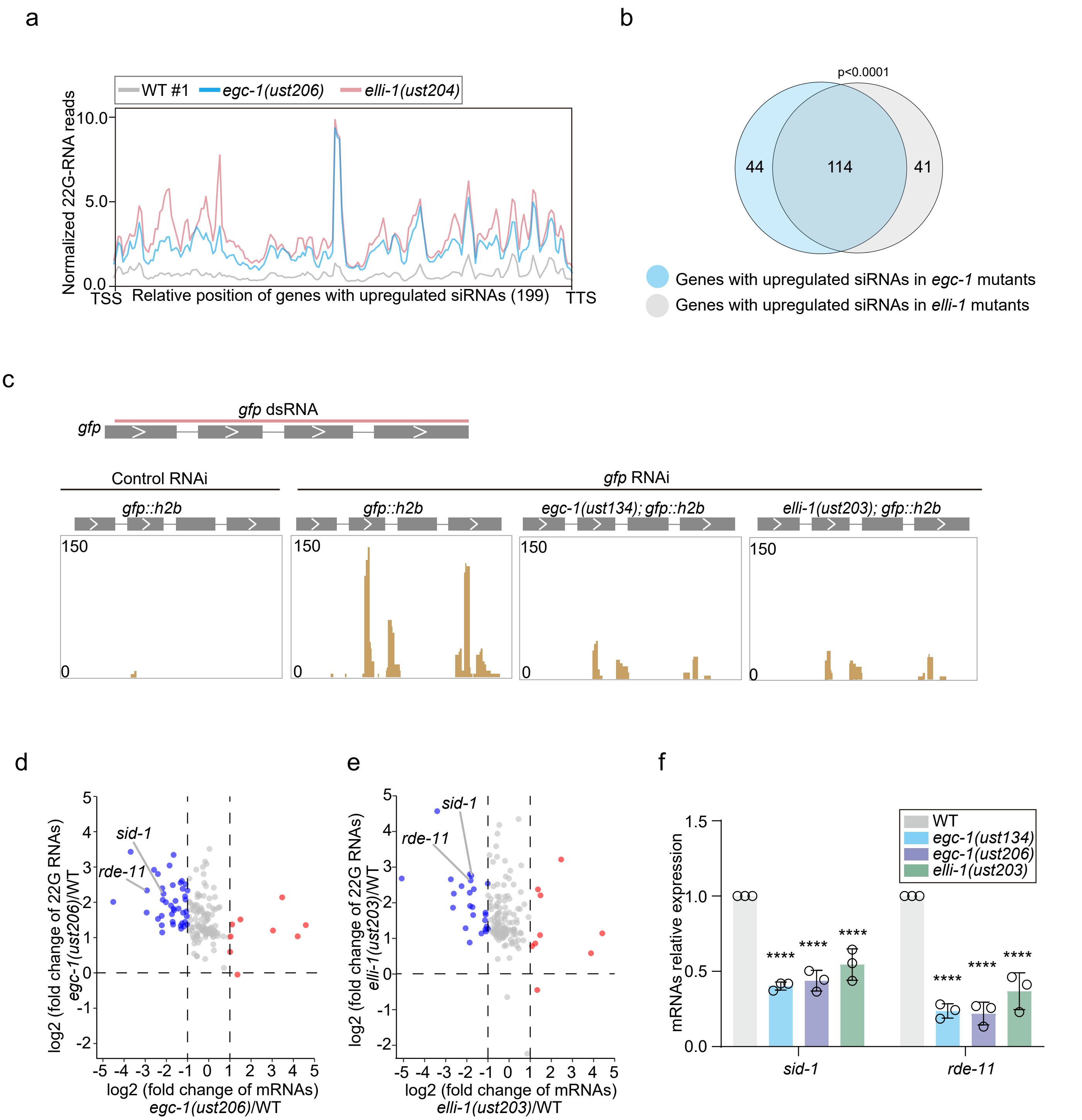

Supplementary Fig. 15. EGC-1 and ELLI-1 coordinate the production of a subset of Mutator foci-derived siRNAs to promote the RNAi response. (a) Metaprofile analysis showing the distribution of normalized 22G RNA (sRNA-seq) reads (RPM) along 199 genes with upregulated siRNAs in *egc-1* or *elli-1* animals in the indicated animals. (b) Proportional venn diagram showing comparison between upregulated siRNAs in *egc-1* and *elli-1* animals. (c) Normalized 22G RNAs mapping to the *gfp* locus in the indicated animals. *gfp::his-58*, *egc-1(-);gfp::his-58* and *elli-1(-);gfp::his-58* animals were fed bacteria expressing *gfp* dsRNAs. Total small RNAs from young adults were extracted and deep-sequenced. (d, e) The volcano plot shows the fold-change of mRNAs (x-axis) versus the fold-change of siRNAs (Y-axis) of the above 199 genes in the indicated animals. *sid-1* and *rde-11* are indicated. (f) mRNA abundances quantified by qRT–PCR in the indicated animals. Data are presented as the mean +/-SD of three biologically independent samples. Statistical analysis was performed with one-way ANOVA with Dunnett's multiple comparison test. ****p < 0.0001. Source data are provided as a Source Data file.

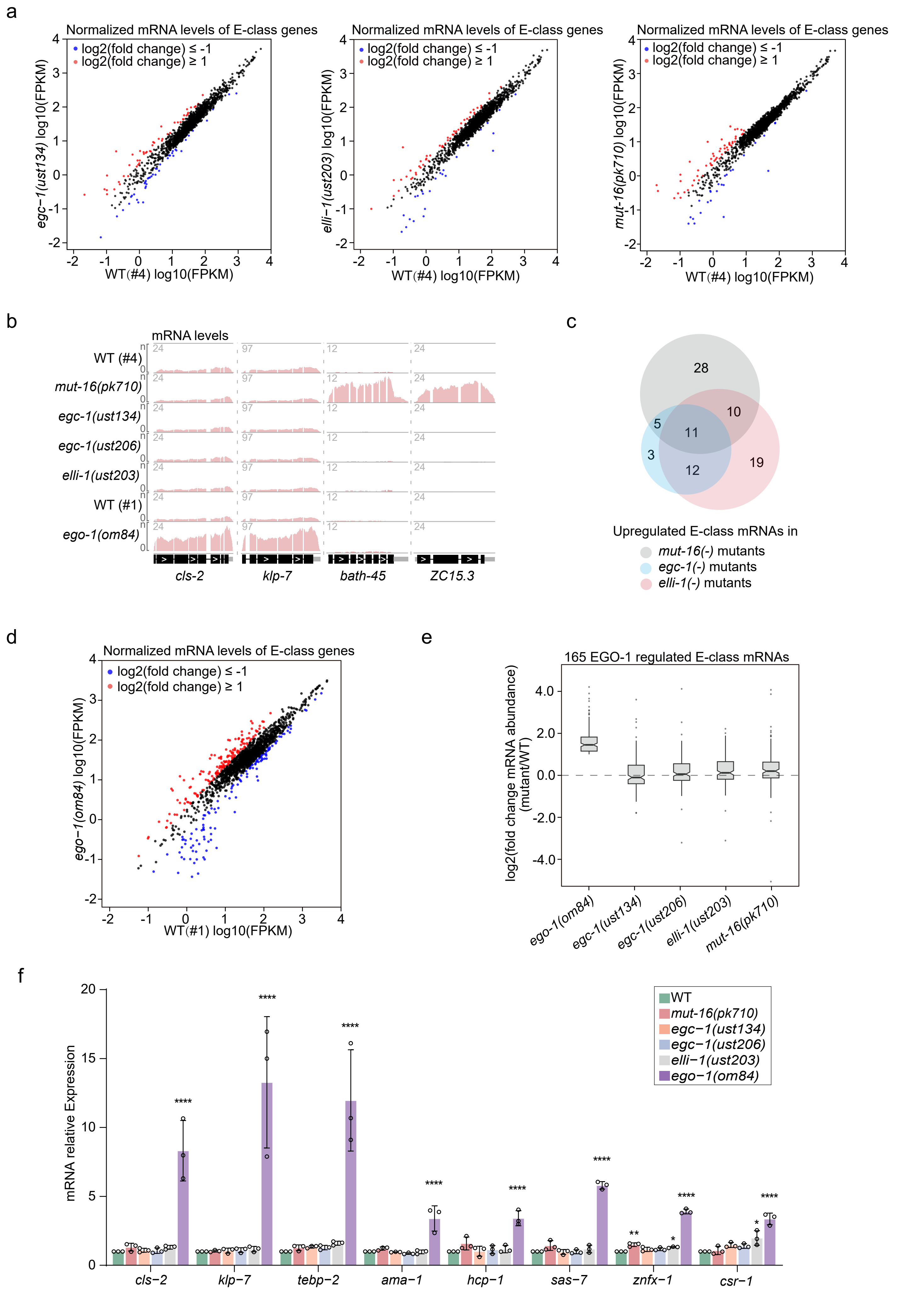

Supplementary Fig. 16. Most E-class genes are not desilenced in *egc-1* or *elli-1* mutants. (legend continued on next page)

Supplementary Fig. 16. Most E-class genes are not desilenced in *egc-1* or *elli-1* mutants. (a) Scatter plots showing normalized mRNA reads (FPKM) mapping to E-class genes in the indicated animals. Genes for which log2(fold change mRNA abundance) ≥1 are colored red, and genes for which log2(fold change mRNA abundance) ≤-1 are colored blue. (b) Normalized mRNA read distribution along *cls-2*, *klp-7*, *bath-45* and *ZC15.3*. *cls-2* and *klp-7* are targets of E-class siRNAs; *bath-45* and *ZC15.3* are targets of M-class siRNAs. (c) Proportional Venn diagram of the overlap between upregulated E-class mRNAs in the indicated animals based on a cutoff criterion of a 2-fold change relative to the level in wild-type animals. (d) Scatter plots showing normalized mRNA reads (FPKM) mapping to E-class genes in wild-type and *ego-1(om84)* animals. Genes for which log2(fold change mRNA abundance) ≥1 are colored red, and genes for which log2(fold change mRNA abundance) ≤-1 are colored blue. (e) Box plot displaying the expression levels of the 165 EGO-1-regulated E-class mRNAs in the indicated animals relative to wild-type animals. Bolded midline indicates median value, box indicates the first and third quartiles, and whiskers represent the most extreme data points within 1.5 times the interquartile range. (f) Quantification of mRNA abundances by qRT–PCR in the indicated animals. Data are presented as the mean +/-SD of three biologically independent samples. Statistical analysis was performed with one-way ANOVA with Dunnett's multiple comparison test. $^{*}p < 0.05$, $^{**}p < 0.01$, $^{****}p < 0.0001$. Source data are provided as a Source Data file.

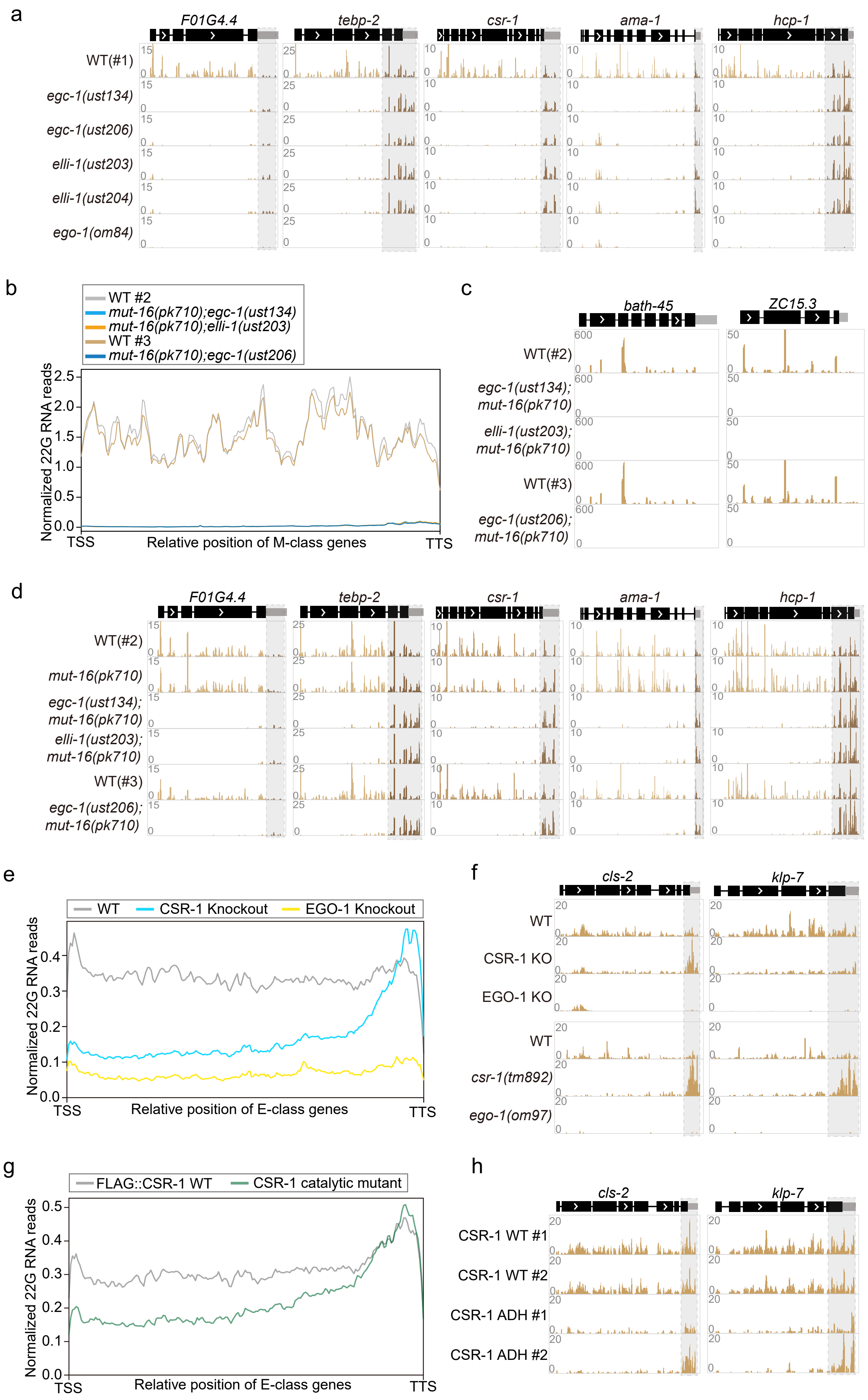

Supplementary Fig. 17. EGC-1, ELLI-1 and CSR-1 promote specialized synthesis of E class 5′ siRNAs.
(legend continued on next page)

Supplementary Fig. 17. EGC-1, ELLI-1 and CSR-1 promote specialized synthesis of E class 5′ siRNAs. (a) Normalized 22G RNA read distribution across E-class siRNA target genes in the indicated animals, including *F01G4.4*, *tebp-2*, *csr-1*, *ama-1* and *hcp-1*. (b) Metaprofile analysis showing the distribution of normalized 22G RNA (sRNA-seq) reads (RPM) along M-class genes in the indicated animals. (c) Normalized 22G RNA read distribution across M-class siRNA target genes in the indicated animals, including *bath-45* and *ZC15.3*. (d) Normalized 22G RNA reads distribution across E-class siRNA target genes in the indicated animals, including *F01G4.4*, *tebp-2*, *csr-1*, *ama-1* and *hcp-1*. Disturbing the assembly of both E and M compartments did not affect the production of E-class 3′ 22G RNAs. (e-h) The deletion of CSR-1, or mutations in CSR-1 that inhibit CSR-1 slicer activity, leads to the loss of 5′ E-class siRNAs without affecting the 3′ E-class siRNAs.Deep-sequence data from published papers were analyzed [10, 15]. (e) Metaprofile analysis showing the distribution of normalized 22G RNA (sRNA-seq) reads (RPM) along E-class genes in the indicated animals. (f) Normalized 22G RNA read distribution across *cls-2* and *klp-7* in the indicated animals. (g) Metaprofile analysis showing the distribution of normalized 22G RNA (sRNA-seq) read (RPM) along E-class genes in the *flag::csr-1* animals and FLAG::CSR-1 catalytic mutants.(h) Normalized 22G RNA read distribution across *cls-2* and *klp-7* in the indicated animals.

Supplementary References:

1. Chen X, *et al.* Dual sgRNA-directed gene knockout using CRISPR/Cas9 technology in *Caenorhabditis elegans*. *Sci Rep-Uk* **4**, (2014).
2. Du Z, *et al.* Condensate cooperativity underlies transgenerational gene silencing. *Cell Rep* **42**, 112859 (2023).
3. Meszaros B, Erdos G, Dosztanyi Z. IUPred2A: context-dependent prediction of protein disorder as a function of redox state and protein binding. *Nucleic Acids Res* **46**, W329-W337 (2018).
4. Price IF, Wagner JA, Pastore B, Hertz HL, Tang W. *C. elegans* germ granules sculpt both germline and somatic RNAome. *Nat Commun* **14**, (2023).
5. Price IF, Hertz HL, Pastore B, Wagner J, Tang W. Proximity labeling identifies LOTUS domain proteins that promote the formation of perinuclear germ granules in *C. elegans*. *Elife* **10**, (2021).
6. Cipriani PG, *et al.* Novel LOTUS-domain proteins are organizational hubs that recruit *C. elegans* Vasa to germ granules. *Elife* **10**, (2021).
7. Wan G, *et al.* ZSP-1 is a Z granule surface protein required for Z granule fluidity and germline immortality in *Caenorhabditis elegans*. *Embo J* **40**, (2021).
8. Placentino M, *et al.* Intrinsically disordered protein PID-2 modulates Z granules and is required for heritable piRNA-induced silencing in the *Caenorhabditis elegans* embryo. *Embo J* **40**, (2021).
9. Maniar JM, Fire AZ. EGO-1, a *C. elegans* RdRP, Modulates Gene Expression via Production of mRNA-Templated Short Antisense RNAs. *Curr Biol* **21**, 449-459 (2011).
10. Claycomb JM, *et al.* The Argonaute CSR-1 and Its 22G-RNA Cofactors Are Required for Holocentric Chromosome Segregation. *Cell* **139**, 123-134 (2009).
11. Gu W, *et al.* Distinct argonaute-mediated 22G-RNA pathways direct genome surveillance in the *C. elegans* germline. *Mol Cell* **36**, 231-244 (2009).
12. Zhang C, *et al.* *mut-16* and other mutator class genes modulate 22G and 26G siRNA pathways in *Caenorhabditis elegans*. *P Natl Acad Sci USA* **108**, 1201-1208 (2011).
13. Manage KI, *et al.* A tudor domain protein, SIMR-1, promotes siRNA production at piRNA-targeted mRNAs in *C. elegans*. *Elife* **9**, (2020).
14. Seroussi U, *et al.* A comprehensive survey of *C. elegans* argonaute proteins reveals organism-wide gene regulatory networks and functions. *Elife* **12**, (2023).
15. Singh M, *et al.* Translation and codon usage regulate Argonaute slicer activity to trigger small RNA biogenesis. *Nat Commun* **12**, (2021).
