# Supplementary material for: Germ granule compartments coordinate specialized small RNA production
Source: Nat Commun. 2024 Jul 10;15:5799. doi: 10.1038/s41467-024-50027-3 (PMC11236994; doi:10.1038/s41467-024-50027-3)
Supplement: Supplementary file 3 — Description of Additional Supplementary Files [file 41467_2024_50027_MOESM3_ESM.pdf]

Description of Additional Supplementary Files

File Name: Supplementary Data 1
Description: A summary table of putative preys identified from EGO-1 Immunoprecipitation Mass Spectrometry (IP-MS) datasets.

File Name: Supplementary Data 2
Description: Lists of E- and M-class genes and siRNA expression patterns in indicated mutants.

File Name: Supplementary Data 3
Description: A list of strains used in this study.

File Name: Supplementary Data 4
Description: A list of qPCR primers used in this study.
