# Supplementary material for: Germ granule compartments coordinate specialized small RNA production
Source: Nat Commun. 2024 Jul 10;15:5799. doi: 10.1038/s41467-024-50027-3 (PMC11236994; doi:10.1038/s41467-024-50027-3)
Supplement: Supplementary file 8 — Reporting Summary [file 41467_2024_50027_MOESM8_ESM.pdf]

nature portfolio

Corresponding author(s): Shouhong Guang

Last updated by author(s): Jun 1, 2024

Reporting Summary

Nature Portfolio wishes to improve the reproducibility of the work that we publish. This form provides structure for consistency and transparency in reporting. For further information on Nature Portfolio policies, see our Editorial Policies and the Editorial Policy Checklist.

Statistics

For all statistical analyses, confirm that the following items are present in the figure legend, table legend, main text, or Methods section.

n/a	Confirmed	
☐	☒	The exact sample size (*n*) for each experimental group/condition, given as a discrete number and unit of measurement
☐	☒	A statement on whether measurements were taken from distinct samples or whether the same sample was measured repeatedly
☐	☒	The statistical test(s) used AND whether they are one- or two-sided<br>*Only common tests should be described solely by name; describe more complex techniques in the Methods section.*
☒	☐	A description of all covariates tested
☒	☐	A description of any assumptions or corrections, such as tests of normality and adjustment for multiple comparisons
☐	☒	A full description of the statistical parameters including central tendency (e.g. means) or other basic estimates (e.g. regression coefficient) AND variation (e.g. standard deviation) or associated estimates of uncertainty (e.g. confidence intervals)
☐	☒	For null hypothesis testing, the test statistic (e.g. *F*, *t*, *r*) with confidence intervals, effect sizes, degrees of freedom and *P* value noted<br>*Give P values as exact values whenever suitable.*
☒	☐	For Bayesian analysis, information on the choice of priors and Markov chain Monte Carlo settings
☒	☐	For hierarchical and complex designs, identification of the appropriate level for tests and full reporting of outcomes
☐	☒	Estimates of effect sizes (e.g. Cohen's *d*, Pearson's *r*), indicating how they were calculated

Our web collection on statistics for biologists contains articles on many of the points above.

Software and code

Policy information about availability of computer code

Data collection	Leica Application Suite X softwares (Version 3.7.2.22383 and Version 3.7.4.23463) were used to acquire images.<br>Small RNAs-seq was performed on an Illumina Hiseq 2500/2000 platform.<br>mRNAs-seq was performed on an Illumina NovaSeq 6000 platform.
Data analysis	Small RNA-seq data was analyzed using the Bowtie2 (V 2.2.5), samtools(V 1.7), python(V 3.8), R (V 4.3.3) and Perl (V 5.18.2).<br>mRNA-seq data was analyzed using the HISAT2 (V 2.1.0), htseq-count(V 2.0.3), Samtools(V 1.7) and R (V 4.3.3).<br>mRNA-qPCR data was analyzed using the GraphPad Prism (V 9.0).<br>IGV (v 2.5.3) was used to visualize the alignment results of siRNAs and mRNAs sequencing data.<br>Quantification of GFP intensities was performed by ImageJ (V 1.8.0) and Pearson’s correlation coefficient was performed by ImageJ2 (V 2.3.0).

For manuscripts utilizing custom algorithms or software that are central to the research but not yet described in published literature, software must be made available to editors and reviewers. We strongly encourage code deposition in a community repository (e.g. GitHub). See the Nature Portfolio guidelines for submitting code & software for further information.

Data

Policy information about availability of data

All manuscripts must include a data availability statement. This statement should provide the following information, where applicable:

- Accession codes, unique identifiers, or web links for publicly available datasets
- A description of any restrictions on data availability
- For clinical datasets or third party data, please ensure that the statement adheres to our policy

The raw sequence data reported in this paper have been deposited in the Genome Sequence Archive in the National Genomics Data Center (China National Center for Bioinformation / Beijing Institute of Genomics, Chinese Academy of Sciences) under accession code CRA013661 [https://ngdc.cncb.ac.cn/gsa/browse/CRA013661] and CRA013663 [https://ngdc.cncb.ac.cn/gsa/browse/CRA013663]. The mass spectrometry proteomics data reported in this paper have been deposited in the OMIX (China National Center for Bioinformation / Beijing Institute of Genomics, Chinese Academy of Sciences) under accession code OMIX006566 [https://ngdc.cncb.ac.cn/omix/release/OMIX006566]. Source data are provided with this paper.

Research involving human participants, their data, or biological material

Policy information about studies with human participants or human data. See also policy information about sex, gender (identity/presentation), and sexual orientation and race, ethnicity and racism.

Reporting on sex and gender	No human research participants were involved.
Reporting on race, ethnicity, or other socially relevant groupings	No human research participants were involved.
Population characteristics	No human research participants were involved.
Recruitment	No human research participants were involved.
Ethics oversight	No human research participants were involved.

Note that full information on the approval of the study protocol must also be provided in the manuscript.

Field-specific reporting

Please select the one below that is the best fit for your research. If you are not sure, read the appropriate sections before making your selection.

☒ Life sciences ☐ Behavioural & social sciences ☐ Ecological, evolutionary & environmental sciences

For a reference copy of the document with all sections, see nature.com/documents/nr-reporting-summary-flat.pdf

Life sciences study design

All studies must disclose on these points even when the disclosure is negative.

Sample size	Sample size are indicated in figure legends or methods. All images are representative of more than three animals. siRNAs from two mutants carrying null alleles of egc-1 and two mutants carrying null alleles of elli-1 were deep sequenced. mRNAs seq data from indicated animals: one biological replicate. qRT-PCR data analysis: three biological replicates. Sample sizes were not predetermined by statistical methods, but by conventional requirements in the respective field.
Data exclusions	No data were excluded from the analysis.
Replication	Phenotype experiments were repeated at least 3 times. Live imaging experiments were performed at least 2 times, and at least 5 germlines were imaged per sample with similar results.
Randomization	For all experiments, control and experimental samples were treated in parallel. Animals were selected randomly from plates for experiments.
Blinding	The investigators were not blind to group allocation during data collection and analysis.

Reporting for specific materials, systems and methods

We require information from authors about some types of materials, experimental systems and methods used in many studies. Here, indicate whether each material, system or method listed is relevant to your study. If you are not sure if a list item applies to your research, read the appropriate section before selecting a response.

Materials & experimental systems

n/a	Involved in the study	
☐	☒	Antibodies
☒	☐	Eukaryotic cell lines
☒	☐	Palaeontology and archaeology
☐	☒	Animals and other organisms
☒	☐	Clinical data
☒	☐	Dual use research of concern
☒	☐	Plants

Methods

n/a	Involved in the study	
☒	☐	ChIP-seq
☒	☐	Flow cytometry
☒	☐	MRI-based neuroimaging

Antibodies

Antibodies used

Anti-GFP (Mouse monoclonal) Abmart Cat# M20004M, Lot number: 314487, Dilution: 1:5000 Western Blot.
Anti-β-ACTIN (Rabbit Monoclonal) Beyotime Cat#: AF5003, Dilution: 1:4000 Western Blot.

Validation

All of these commercial purchased antibodies were validated by the manufacturer or in our previous studies, more specially: Anti-GFP antibody has been validated by Abmart by demonstrating Western blot (http://www.ab-mart.com.cn/page.aspx?node=%2059%20&id=%20971); Anti-β-ACTIN antibody was validated in our previous works (Huang, et al, 2022 and Xu, et al, 2023).

Animals and other research organisms

Policy information about studies involving animals; ARRIVE guidelines recommended for reporting animal research, and Sex and Gender in Research

Laboratory animals

Embryos, larvae, and adults of Caenorhabditis elegans were used.

Wild animals

No wild animals were used in this study.

Reporting on sex

Hermaphrodite animals were used in this study.

Field-collected samples

No field-collected samples were used in this study.

Ethics oversight

Caenorhabditis elegans was used in this study and no ethical approval was required.

Note that full information on the approval of the study protocol must also be provided in the manuscript.

Plants

Seed stocks

Report on the source of all seed stocks or other plant material used. If applicable, state the seed stock centre and catalogue number. If plant specimens were collected from the field, describe the collection location, date and sampling procedures.

Novel plant genotypes

Describe the methods by which all novel plant genotypes were produced. This includes those generated by transgenic approaches, gene editing, chemical/radiation-based mutagenesis and hybridization. For transgenic lines, describe the transformation method, the number of independent lines analyzed and the generation upon which experiments were performed. For gene-edited lines, describe the editor used, the endogenous sequence targeted for editing, the targeting guide RNA sequence (if applicable) and how the editor was applied.

Authentication

Describe any authentication procedures for each seed stock used or novel genotype generated. Describe any experiments used to assess the effect of a mutation and, where applicable, how potential secondary effects (e.g. second site T-DNA insertions, mosiacism, off-target gene editing) were examined.
